# Hyaluronan-CD44 Interaction Regulates Mouse Retinal Progenitor Cells Migration, Proliferation and Neuronal Differentiation

**DOI:** 10.1007/s12015-023-10622-1

**Published:** 2023-09-14

**Authors:** Jian Ma, Xiaoyun Fang, Min Chen, Yao Wang, Li Zhang

**Affiliations:** Eye Center, the Second Affiliated Hospital, Zhejiang University School of Medicine, Hangzhou, 310009 China

**Keywords:** Retinal progenitor cells, Migration, Proliferation, Differentiation, Hyaluronan-CD44

## Abstract

Cell-based therapies have shown great potential because of their abilities to replace dying retinal neuron cells and preserve vision. The migration, proliferation and differentiation of retinal progenitor cells(RPCs) plays a vital role in the integration of the RPCs into the retina when transplanted into the host. Our study aimed to explore the effects of Hyaluronan(HA)-CD44 interactions on the regulation of RPCs migration, proliferation and differentiation, and investigate the underlying regulatory mechanisms. We found that CD44 was expressed in RPCs, and the HA-CD44 interaction markedly improved RPCs adhesion and migration. The stimulation of microRNA-21(miR-21) expression by the HA-CD44 interaction was protein kinase C (PKC)/Nanog-dependent in RPCs. Treatment of RPCs with PKC- or Nanog-specific ASODN or miR-21 antagomir effectively blocked HA-mediated RPCs adhesion and migration. Moreover, Rho-Kinase(ROK)/ Grb2-associated binders(Gab-1) associated phosphatidylinositol 3-kinase(PI3K)/AKT signalling activation was required for HA-CD44 interaction mediated RPCs proliferation and neuronal differentiation. Our findings demonstrated new roles for the HA-CD44 interaction in regulating the migration, proliferation and neuronal differentiation of RPCs. HA-CD44 signalling could represent a novel approach to controlling RPC fates, and the findings may be instructive for the application of RPCs for future therapeutic applications.

**Graphical Abstract:**

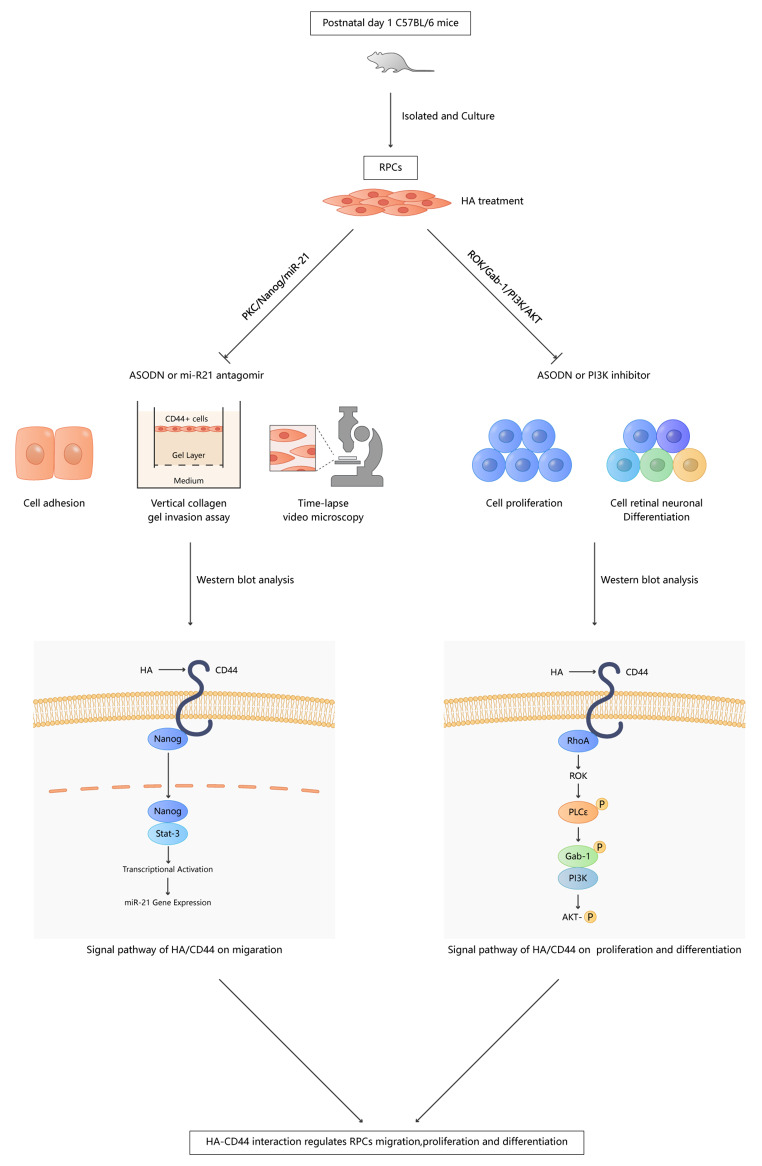

**Supplementary Information:**

The online version contains supplementary material available at 10.1007/s12015-023-10622-1.

## Introduction

Inherited and age-related retinal degeneration(AMD) are the leading cause of currently untreatable blindness worldwide. Almost 30 million people worldwide are affected by retinal degeneration including AMD and retinitis pigmentosa(RP). Pharmacological treatments anti-vascular endothelial growth factor(VEGF) could save the eyesight of exudative AMD patients, and gene therapies with Luxturn can delay the process of inherited retinal degeneration caused by mutations in the RPE65 gene. However, there are no FDA-approved gene- or stem cell-based therapies yet that target the genetic subtypes of AMD, or exudative AMD. Furthermore in many cases, the irreversible loss of photoreceptors in later stages would make gene therapy ineffective [1]. Currently, cell-based therapies have shown great potential because of their abilities to replace dying retinal neuron cells and preserve vision [2, 3]. Two main cell-based mechanistic approaches are being tested in clinical trials. Replacement therapies utilize cell-derived retinal pigment epithelial (RPE) cells or photoreceptor cells to supplant lost or defective host RPE or photoreceptor cells. Preservation therapies utilize supportive cells to aid in visual function and photoreceptor preservation partially by neurotrophic mechanisms. The ideal cell source for treatments must meet some criteria. Firstly, the cells would need to be easily propagated and maintained. Secondly, derivation and differentiation protocols would need to be optimized for efficient and reproducible cell lines for large scale production. Thirdly, the cells would need to be highly efficacious with no tumor formation or immunologic response [2]. Till now a range of cells have been adopted involving neural progenitor cells(NPCs), retinal progenitor cells(RPCs), mesenchymal stem cells(MSCs), embryonic stem cells(ESCs), induced pluripotent stem cells(iPSCs) [4]. Among these cells, retinal progenitor cells (RPCs) capable of self-renewal and differentiation into various retinal cell types have received attention for sight restoration [5, 6]. RPCs were discovered in the adult mammalian eye and successfully isolated from the human retina, and they can restore impaired visual function without tumorigenicity and ethical concerns [5, 7]. The migration, proliferation and differentiation of RPCs plays a vital role in the integration of the RPCs into host when transplanted into recipient mice, rats or other non-human primates. There are lots of methods optimizing the transplantion including using biomaterials like mussel-inspired injectable hydrogel or rapid photopolymerization-mediated production of PCL-based bioabsorbable scaffold [8, 9]. Therefore, it is extremely important to explore the mechanisms that control RPCs migration and differentiation.

Hyaluronic acid (HA) is the simplest glycosaminoglycan and a major component of the extracellular matrix (ECM) [10]. Our recent study showed that chondroitinase ABC facilitated the migration of mouse RPCs via the disruption of glial barriers, and HA/CD44 signalling pathway action may be the mechanism underlying the effect [11]. Accumulating evidence has demonstrated that HA plays a role in many facets of stem cell biology [12]. Previous studies have shown that HA supports mouse RPCs growth in vitro and in vivo [10]. HA not only regulates cell adhesion and motility, but also mediates cell proliferation and differentiation [8]. CD44, a major cell surface receptor for HA, is a family of multifunctional transmembrane glycoproteins that is expressed in numerous cells and tissues, including stem cells [13]. As reported, CD44 can connect the extracellular matrix to the cellular cytoskeleton and coordinate multiple downstream signalling pathways [13–15]. Recent studies have indicated that the HA-CD44 interaction promotes both the growth and invasion of head and neck squamous cell carcinoma [13]. However, the role of the HA-CD44 interaction in the regulation of RPCs migration, proliferation and differentiation remains unknown.

In this study, the role of the HA-CD44 interaction in the migration, proliferation and differentiation of RPCs was investigated. We observed that CD44 was expressed in mouse RPCs and further identified that the HA-CD44 interaction increased RPCs migration via PKC/Nanog/miR-21 signalling and promoted the proliferation and retinal neuronal differentiation of RPCs via ROK/Gab-1 and PI3K/AKT signalling. Our findings provide important new insights into the mechanisms by which the HA-CD44 interaction regulates RPCs migration, proliferation and differentiation and could be used to develop a new strategy for improving the repair result of RPCs in future therapeutic applications for RD.

## Materials and Methods

### RPCs Isolation and Culture

According to our previous studies [5, 11, 16], RPCs were obtained from fresh retinal tissue of postnatal day 1 C57BL/6 mice, and then were cultured with proliferation medium containing advanced Dulbecco’s modified Eagle’s medium (DMEM)/F12 (Invitrogen, Carlsbad, CA, USA), 20 ng/ml recombinant epidermal growth factor (EGF, Invitrogen), 2 mM L-glutamine (Invitrogen) and 1% N2 neural supplement (Invitrogen). For the differentiation study, RPCs were cultured with differentiation medium containing advanced DMEM/F12 (Invitrogen), 10% fetal bovine serum (FBS, Invitrogen), 1% N2 neural supplement (Invitrogen) and without EGF.

RPCs morphology was assessed by scanning electron microscopy. All animal experiments were approved by the Animal Ethics Committee of the Second Affiliated Hospital, School of Medicine, Zhejiang University, and performed in compliance with the ARRIVE guidelines. The IRB number is 2021 − 0155.

### Drug Treatment

RPCs were treated with HA (100 µg/ml) for 72 h or CD44 antibody (10 µg/ml) for 3 h followed by HA (100 µg/ml) for 72 h to assess the effect of the HA-CD44 interaction on cell migration, proliferation and differentiation. For the knockdown experiments, cells were transfected with antisense oligonucleotides (ASODNs) or sense oligonucleotides (SODNs), miR-21 inhibitor or negative control. Sequences of antisense oligonucleotides and sense oligonucleotides are provided in Table 1.

**Table 1:** Sequences of antisense oligonucleotides and sense oligonucleotides

	ASOND 5’-3’	SODN 5’-3’
PKC	TAATATATCTGATAGA GTGCCAGTG	CACTGGCACTCTATCAG ATATATTA
Nanog	TAACACACCAAGTTG TAAATAGAGC	GCTCTATTTACAACTTG GTGTGTTA
Gab-1	UAAUGUUUAACACA UAAACAG	GUUUAUGUGUUAAACA UUAUC
ROK	AAUAACAGCAAUCU UAACCCU	GGUUAAGAUUGCUGUU AUUCU

### Transfection

ASODNs and SODNs were synthesized with a phosphorothioate backbone, and purified with ULTRAPAGE (Sangon, Inc. Shanghai, China). For transient transfection, RPCs were treated with appropriate concentrations of ODNs, miR-21 inhibitor, or negative control using Lipofectamine 2000 Reagent (Invitrogen) according to the manufacturer’s instructions for 4 h. Then, the medium was removed and replaced with proliferation or differentiation medium.

### Cell Adhesion Assay

The cell adhesion assay was performed as previously described with several modifications [17]. The 96-well plates were treated overnight with Matrigel (0.04 mg/mL) (BD Biosciences, San Jose, CA) to facilitate cell attachment. The different groups of RPCs were trypsinized and added to each well, allowed to attach for 2 h, and then washed gently with PBS twice. MTT solution (5 mg/mL) was added to the cells, and plates were further incubated at 37 ℃ for 4 h. The supernatant was carefully removed, and dimethyl sulfoxide (DMSO; Sigma-Aldrich, St. Louis, MO) was added to dissolve formazan crystals. The optical density was read on a spectrophotometer (Sunrise RC, Tecan, Switzerland) through a 490 nm filter. Cell adhesion rates = (OD of the treated group cells/OD of the control group cells) x 100%.

### Western Blot Analysis

Proteins were isolated from the cultured RPCs, which were extracted in RIPA solution (Beyotime, Shanghai, China) with a protease inhibitor cocktail (Roche) and their concentrations were determined by the BCA protein assay kit (Beyotime; Beijing, China). Next, an equal amount of protein (50 µg) from each sample was separated via sodium dodecyl sulfate-polyacrylamide gel electrophoresis (SDS-PAGE), and then transferred to polyvinylidene difluoride (PVDF) membranes (Millipore; Billerica, MA, USA). The membranes were blocked and incubated with primary antibodies against the following molecules overnight at 4 °C: HA, PKC, β-tubulin (Abcam), CD44, Nanog, eIF4A, survivin, PDCD4, β-actin (Proteintech), MDR1, XIAP (Abgent), ROK, p-Gab-1, Gab-1, p-AKT, AKT, cyclin D1 (Affinity), Hes1 (Saierbio) and GAPDH. The bands were detected with a chemiluminescence reagent and imaged by the ChemiDoc MP System (Bio-Rad; Hercules, CA, USA). The bands’ intensities were quantified using Image Laboratory (version 2.0) software.

### Flow Cytometry

At passages 2 and 4, 1 × 10^6^ RPCs were collected. CD44 expression on the RPCs was evaluated by flow cytometry using fluorescein conjugated HA (FL-HA) as previously described [18].

### Vertical Collagen Gel Invasion Assay

The vertical collagen gel invasion assay was performed as previously described [19]. Monocyte chemoattractant protein-1 (MCP-1) was poured in the first layer as a chemotactic reagent, and human umbilical vein endothelial cells (HUVECs) labelled with cell tracker red dye (Invitrogen, Carlsbad, CA, USA) were seeded onto the top surface of collagen gel and incubated at 37 °C in 5% CO_2_ for 2 h to form a confluent monolayer. The different groups of RPCs labelled with cell tracker green dye (Invitrogen, Carlsbad, CA, USA) were added onto HUVECs and incubated at 37 °C in 5% CO_2_ for 24 h in the vertical position. The migration of cells was measured as the maximum distance from the surface of the collagen gel under fluorescence microscopy.

### Time-Lapse Video Microscopy

During the time-lapse recording, the different groups of RPCs were maintained in a chamber at 37 °C with a 5% CO_2_ atmosphere. To analyse migration behaviour, serial phase-contrast images were captured with an inverted microscope (Zeiss Axiovert 200 M) at 30 s intervals. The images were built into a movie using Metamorph software.

### Immunocytochemistry

At the determined times, the different groups of RPCs were fixed with 4% paraformaldehyde (PFA). After blocking with 10% normal goat serum (Sigma-Aldrich), the cells were incubated with one of the following primary antibodies: Ki67 (1:50), β-III-tubulin (1:500), Recoverin (1:200), Rhodopsin (1:500, Abcam) and Crx (1:50, Omnimabs) at 4 °C overnight. Then, they were labelled with the corresponding secondary antibodies: Alexa 488-conjugated anti-mouse, Cy3-conjugated anti-mouse or FITC-conjugated anti-rabbit antibodies (1:500, Jackson). Finally, the cells were counterstained with DAPI nuclear stain and observed by fluorescence microscopy (Leica, Germany). The positive ratio was calculated with (immunopositive cells/DAPI stained cells in the field) × 100% by ImageJ software.

### Cell Proliferation Assay

The cell proliferation assay was performed using a cell counting kit (CCK-8, Dojindo, Kumamoto, Japan) according to the instructions [20]. The different groups of RPCs were incubated with CCK-8 solution for 4 h, and the absorbance at 450 nm was read using a spectrophotometer (ELX800, BioTek, Vermont, USA).

### Reverse Transcription and Quantitative Polymerase Chain Reaction (qPCR)

Total RNA was extracted using Trizol Reagent (Invitrogen) and reverse transcribed according to the manufacturer’s instructions. Then, qPCR was performed using specific primers (Table 2). Relative miRNA expression was normalized to that of U6 (2^−∆Ct^).

**Table 2:** qPCR primers for miRNAs

miRNA	Primer
mmu miR21	TGCGGCTAGCTTATCAGACT
mmu miR191	AGCAGGTGCGGGGCGGCGAAA
mmu miR universal	CCAGTCTCAGGGTCCGAGGTATTC
mmu U6	CTCGCTTCGGCAGCACA

### Statistical Analysis

Statistical results were expressed as the mean ± SD. The data were statistically analysed by one-way analysis of variance (ANOVA) or Student’s *t-test*, and *P* ≤ 0.05 was considered statistically significant.

## Results

### RPCs Express CD44 in Vitro

**Fig. 1:**
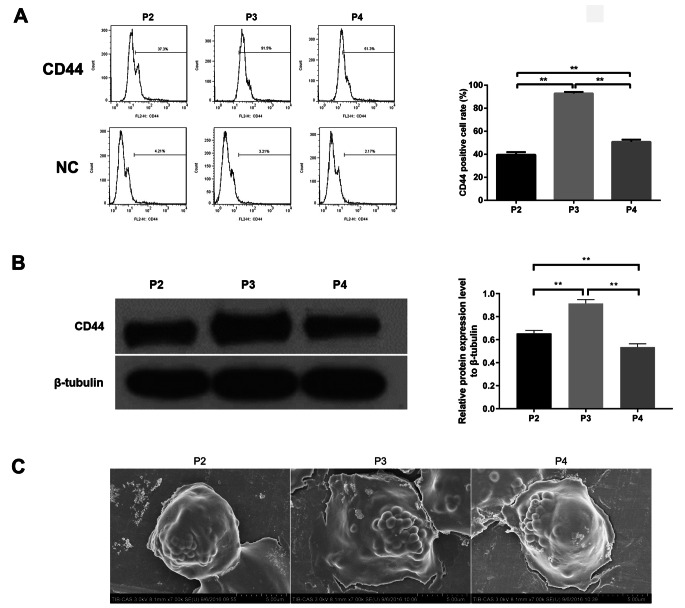
Expression of CD44 on RPCs in vitro. **(A)** Flow cytometry was used to analyse CD44 expression from P2 to P4 RPCs (n = 4). **(B)** Western blot results showed the CD44 expression from P2 to P4 RPCs (n = 3). **(C)** RPCs morphology was assessed by scanning electron microscopy. The data are shown as the mean ± SD, **p < 0.01

To investigate the role of CD44 in RPCs migration and differentiation, we first explored the expression of CD44 in RPCs by flow cytometry and western blot analysis. As shown in Fig. 1, flow cytometry analysis showed that CD44 was detected in P2, P3, and P4 RPCs, with more significant expression in P3 RPCs. This result was strongly confirmed by western blot analysis, and CD44 expression in P3 RPCs was significantly greater than that in P2 or P4 RPCs. The morphology of RPCs was assessed by scanning electron microscopy. These findings strongly indicated that RPCs express CD44 in vitro.

### HA-CD44 Interaction Increases RPCs Adhesion and Migration via PKC/Nanog/miR-21 Signalling

**Fig. 2:**
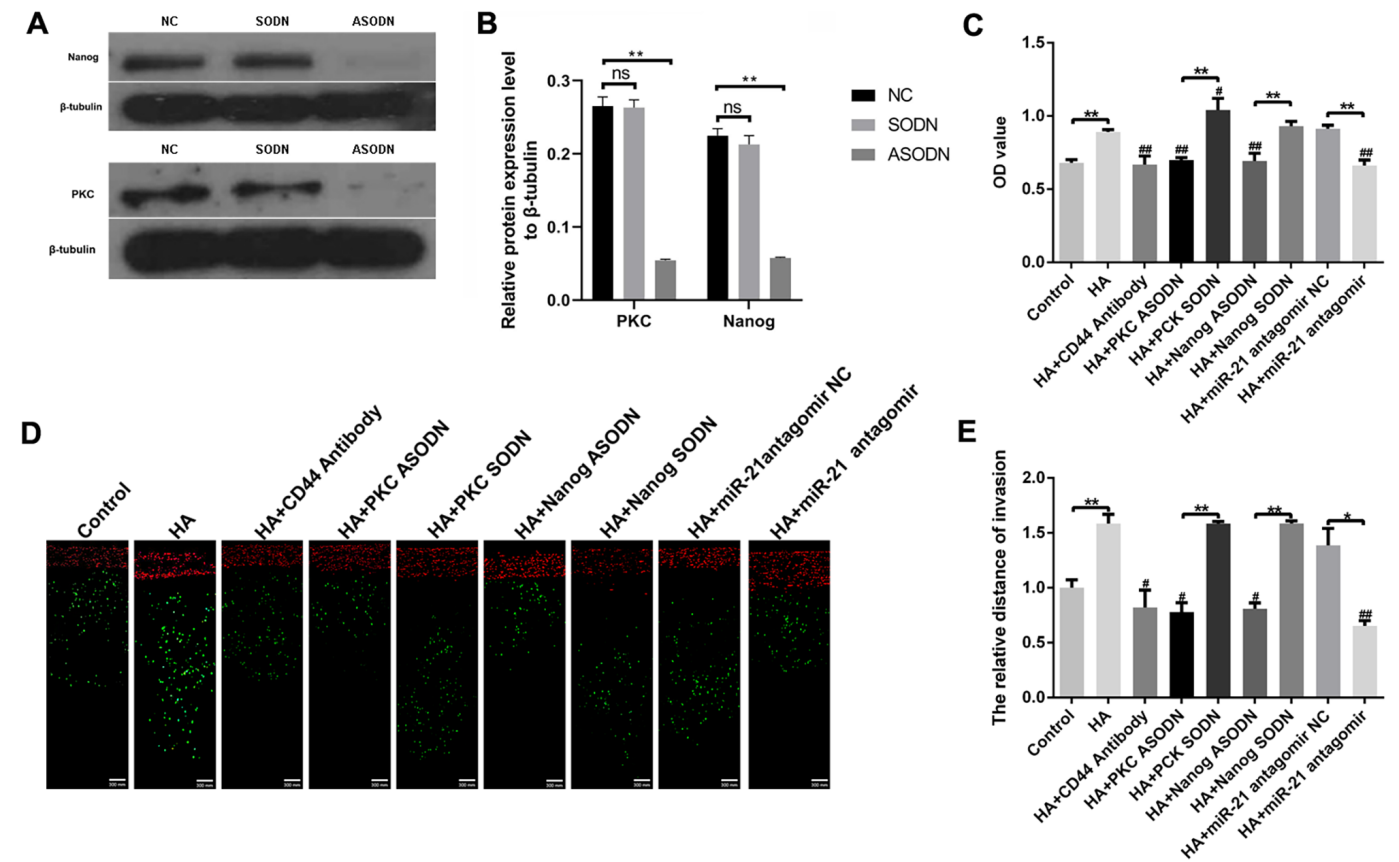
HA-CD44 interaction and PKC/Nanog/miR-21 signalling are closely linked to RPCs adhesion and migration. **(A, B)** Western blot results revealed that the expression levels of PKC and Nanog were significantly decreased in the PKC- and Nanog-specific ASODN-transfected cells compared with the control SODN-transfected cells (n = 3 ). **(C)** Cell adhesion ability of the RPCs with different treatments was assessed via MTT analysis (n = 3). **(D, E)** Cell migration ability of RPCs with different treatments was assessed using a transendothelial assay (n = 3 ). The data are shown as the mean ± SD, *p < 0.05; **p < 0.01; ^#^*p* < 0.05, ^##^*p* < 0.01 versus the HA group

The HA-CD44 interaction plays a pivotal role in cytoskeleton activation and cell migration [21]. Previous studies indicated that HA/CD44-mediated PKC signalling regulates stem cell marker (Nanog)-associated miR-21 production, which in turn downregulates the tumour suppressor protein (PDCD4) and promotes oncogenesis, thus leading to survivin, X-linked inhibitor of apoptosis protein (XIAP) and MDR1 expression. Survivin, XIAP and MDR1 play key roles in cell survival, expansion, and migration [22]. In this study we focused on whether the HA-CD44 interaction regulates RPCs migration via PKC/Nanog/miR-21 signalling. To confirm the involvement of the HA-CD44 interaction and PKC/Nanog/miR-21 signalling in RPCs adhesion and migration, CD44 or PKC/Nanog/miR-21 signalling was inhibited. We first determined the effect of PKC- and Nanog-specific ASODNs on PKC and Nanog protein levels. As expected, PKC and Nanog protein levels were significantly decreased in PKC- and Nanog-specific ASODN-transfected cells compared with control SODN-transfected cells (Fig. 2A and B).

The results of an MTT assay for cell adhesion are shown in Fig. 2C. The adhesion rate was greater in RPCs treated with HA than in the control or pretreated with anti-CD44 antibody followed by HA addition. Further analyses indicate that RPCs treated with PKC-specific ASODN or Nanog-specific ASODN display a decreased adhesion rate in the presence of HA. We also noted that downregulation of miR-21 by treating RPCs with miR-21 antagomir induced a significant decline in the cell adhesion rate of RPCs in the presence of HA. Cell migration assays were conducted using a transendothelial assay and time-lapse imaging. Our data showed that RPCs treated with HA exhibited better ability to migrate than control or RPCs pretreated with anti-CD44 antibody followed by HA addition (Fig. 2D and E). We also noted that RPCs treated with PKC-specific ASODN, Nanog-specific ASODN or miR-21 antagomir displayed a decreased ability to migrate in the presence of HA (Fig. 2D and E). Time-lapse imaging results were consistent with the results shown above (shown in Supplementary Material).

**Fig. 3:**
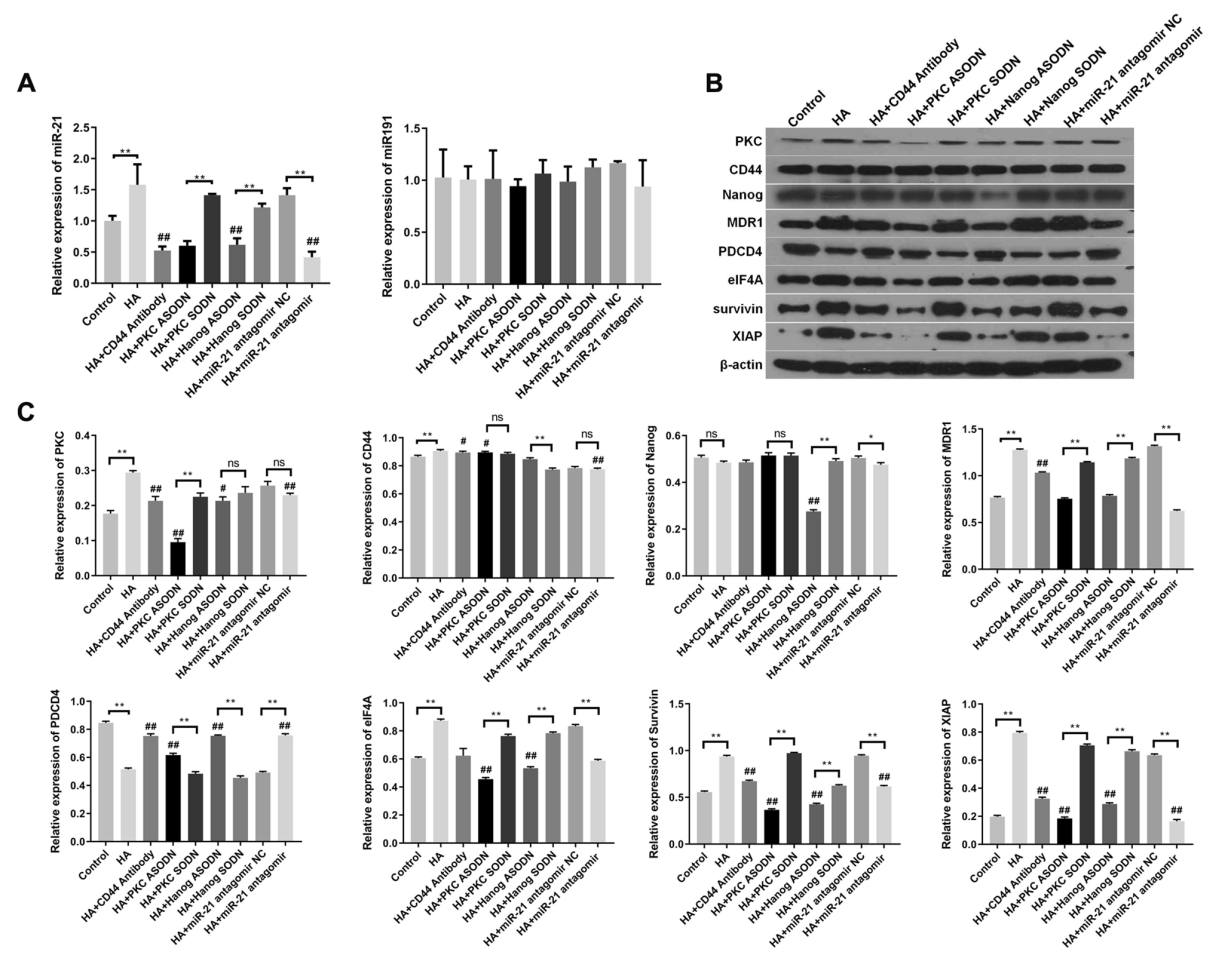
HA-CD44 interaction activates PKC/Nanog/miR-21 signalling and regulates the expression of migration-related proteins in RPCs. **(A)** Expression levels of miR-21 and miR-191 with different treatments were analysed by qPCR (n = 3). **(B, C)** Expression levels of CD44, PKC, Nanog and migration-related proteins with different treatments were analysed by western blot (n = 3). The data are shown as the mean ± SD, *p < 0.05; **p < 0.01; ^#^*p* < 0.05, ^##^*p* < 0.01 versus the HA group

To determine whether miR-21 levels are upregulated following the binding of HA to CD44, our results indicate that the level of miR-21 is increased in RPCs treated with HA (Fig. 3A) compared with control or those cells pretreated with anti-CD44 antibody followed by HA treatment (Fig. 3A). These findings suggest that the HA-CD44 interaction has an important role in the production of miR-21 in RPCs. Furthermore, RPCs treated with PKC-specific ASODN or Nanog-specific ASODN showed significantly less HA-induced miR-21 expression than the SODN groups (Fig. 3A). These findings support the notion that both PKC and Nanog are required for miR-21 production in HA-activated RPCs. In addition, we found that the expression of miR-21 was induced in RPCs treated with miR-21 antagomir NC upon the addition of HA (Fig. 3A). In contrast, the treatment of RPCs with miR-21 antagomir plus HA resulted in a decrease in miR-21 expression (Fig. 3A). Moreover, significant differences were not observed among the levels of the miR-191 control in all samples (Fig. 3A).

Finally, we explored HA/CD44-mediated PKC/Nanog/miR-21 signalling on the expression of MDR1, eIF4A, survivin, XIAP and PDCD4. Our results indicate that significant increases in PKC, MDR1, eIF4A, survivin and XIAP expression occurred in RPCs treated with HA compared with RPCs treated with no HA (Fig. 3B) or pretreated with anti-CD44 antibody followed by HA addition (Fig. 3C). In contrast, HA treatment promoted the downregulation of the PDCD4 expression in RPCs (Fig. 3C). Furthermore, we observed that significant inhibition of MDR1, eIF4A, survivin and XIAP expression occurred in RPCs pretreated with PKC-specific ASODN or Nanog-specific ASODN but not SODN followed by HA addition (Fig. 3C). We also confirmed that the downregulation of miR-21 by antagomir promotes downregulation of MDR1, eIF4A, survivin and XIAP in the presence of HA (Fig. 3C). However, significant increases in PDCD4 expression was observed when PKC/Nanog/miR-21 signalling was inhibited.

These observations confirm that the HA-CD44 interaction and PKC/Nanog/miR-21 signalling are closely linked to RPCs adhesion and migration.

### HA-CD44 Interaction Promotes Proliferation and Retinal Neuronal

#### Differentiation of RPCs via ROK/Gab-1 and PI3K/AKT Signalling

**Fig. 4:**
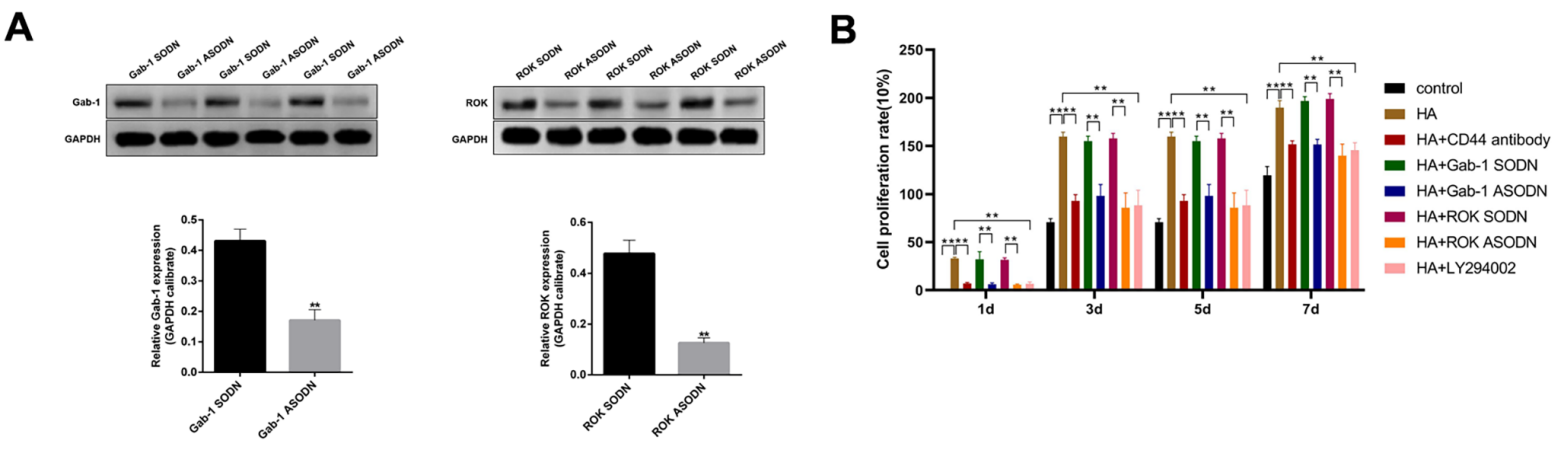
HA-CD44 interaction promotes RPCs proliferation via ROK/Gab-1 and PI3K/AKT signalling. **(A)** Western blot results revealed that the expression levels of Gab and ROK were significantly decreased in the Gab- and ROK-specific ASODN-transfected cells compared with the control SODN-transfected cells (n = 3). **(B)** Proliferation of RPCs with different treatments was analysed by CCK-8 assay (n = 3). The data are shown as the mean ± SD, ^**^p < 0.01; ***p < 0.001; scale bar = 50 μm

ROK/Gab-1 and PI3K/AKT are key signalling pathways in HA/CD44-mediated cellular functions, such as proliferation and cell survival [13]. Previous work has also indicated a positive link between ROK/Gab-1 associated PI3K/AKT signalling activation during HA/CD44-mediated breast cancer progression [13]. In this study, we focused on whether the HA-CD44 interaction regulates the proliferation and retinal neuronal differentiation of RPCs via ROK/Gab-1 and PI3K/AKT signalling. To confirm the involvement of the HA-CD44 interaction and ROK/Gab-1 and PI3K/AKT signalling in RPCs proliferation and differentiation, CD44 or ROK/Gab-1 and PI3K/AKT signalling was inhibited. We first determined the effect of Gab-1- and ROK-specific ASODN on Gab-1 and ROK protein levels. As expected, Gab-1 and ROK protein levels were significantly decreased in Gab-1- and ROK-specific ASODN-transfected cells compared with control SODN-transfected cells (Fig. 4A).

**Fig. 5:**
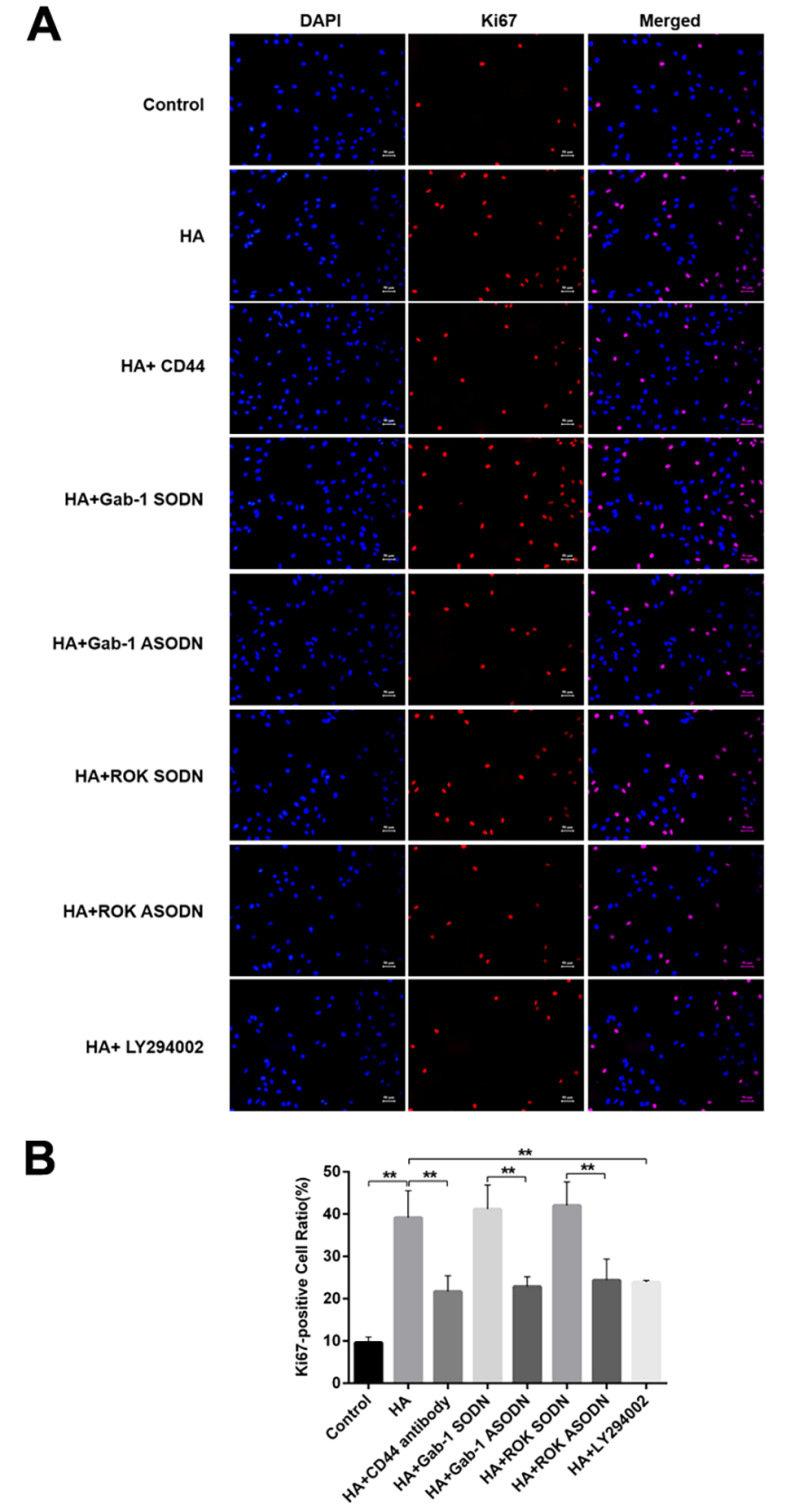
HA-CD44 interaction promotes RPCs proliferation via ROK/Gab-1 and PI3K/AKT signalling. **(A, B)** Proliferation of RPCs with different treatments was analysed by Ki67 immunofluorescence assay (n = 3). The data are shown as the mean ± SD, ^**^p < 0.01; scale bar = 50 μm

The results of the CCK-8 assay showed that the proliferation of RPCs treated with HA was significantly increased compared with that of the control or pretreated with anti-CD44 antibody followed by HA addition (Fig. 4B). Further analyses indicated that RPCs treated with ROK-specific ASODN, Gab-1-specific ASODN or the PI3K inhibitor LY294002 displayed a decreased proliferation rate in the presence of HA (Fig. 4B). At the same time, the immunofluorescence assay results showed increased expression levels of Ki67 in RPCs treated with HA compared with the control or pretreated with anti-CD44 antibody followed by HA addition (Fig. 5A, B). Similarly, the expression levels of Ki67 were reduced in RPCs treated with ROK-specific ASODN, Gab-1-specific ASODN or LY294002 (Fig. 5A, B). These data indicated that the HA-CD44 interaction regulates proliferation of RPCs via ROK/Gab-1 and PI3K/AKT signalling.

**Fig. 6:**
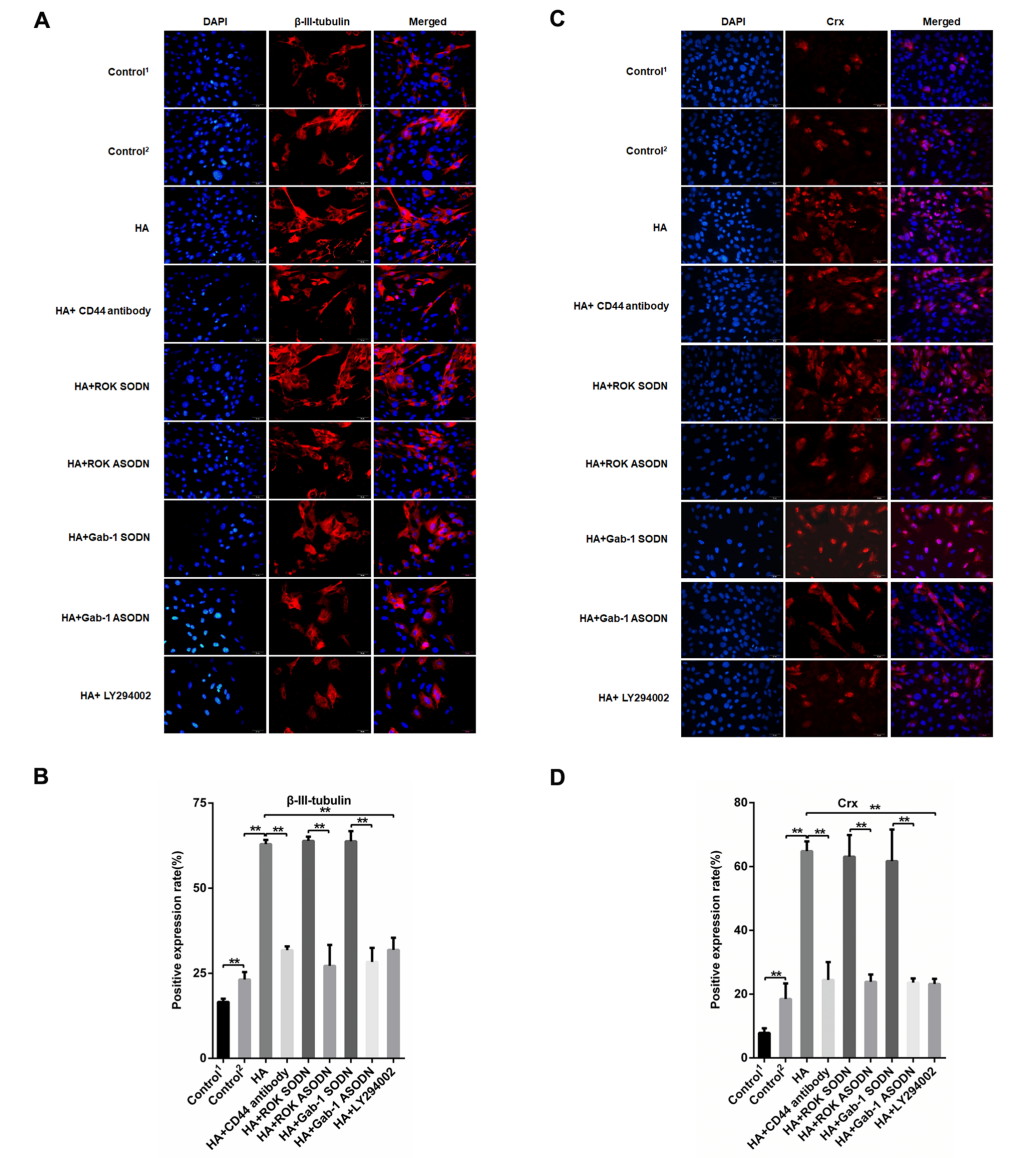
HA-CD44 interaction, ROK/Gab-1 and PI3K/AKT signalling enhance the differentiation of RPCs towards neuronal cells. (A, B) Immunocytochemistry analysis was used to detect β-III-tubulin-positive cells with different treatments (n = 3). (C, D) Immunocytochemistry analysis was used to detect cone-rod homeobox (Crx)-positive cells with different treatments (n = 3). The data are shown as the mean ± SD, ^**^p < 0.01; Scale bar = 20 mm; Control^1^, proliferation medium, Control^2^, differentiation medium

**Fig. 7:**
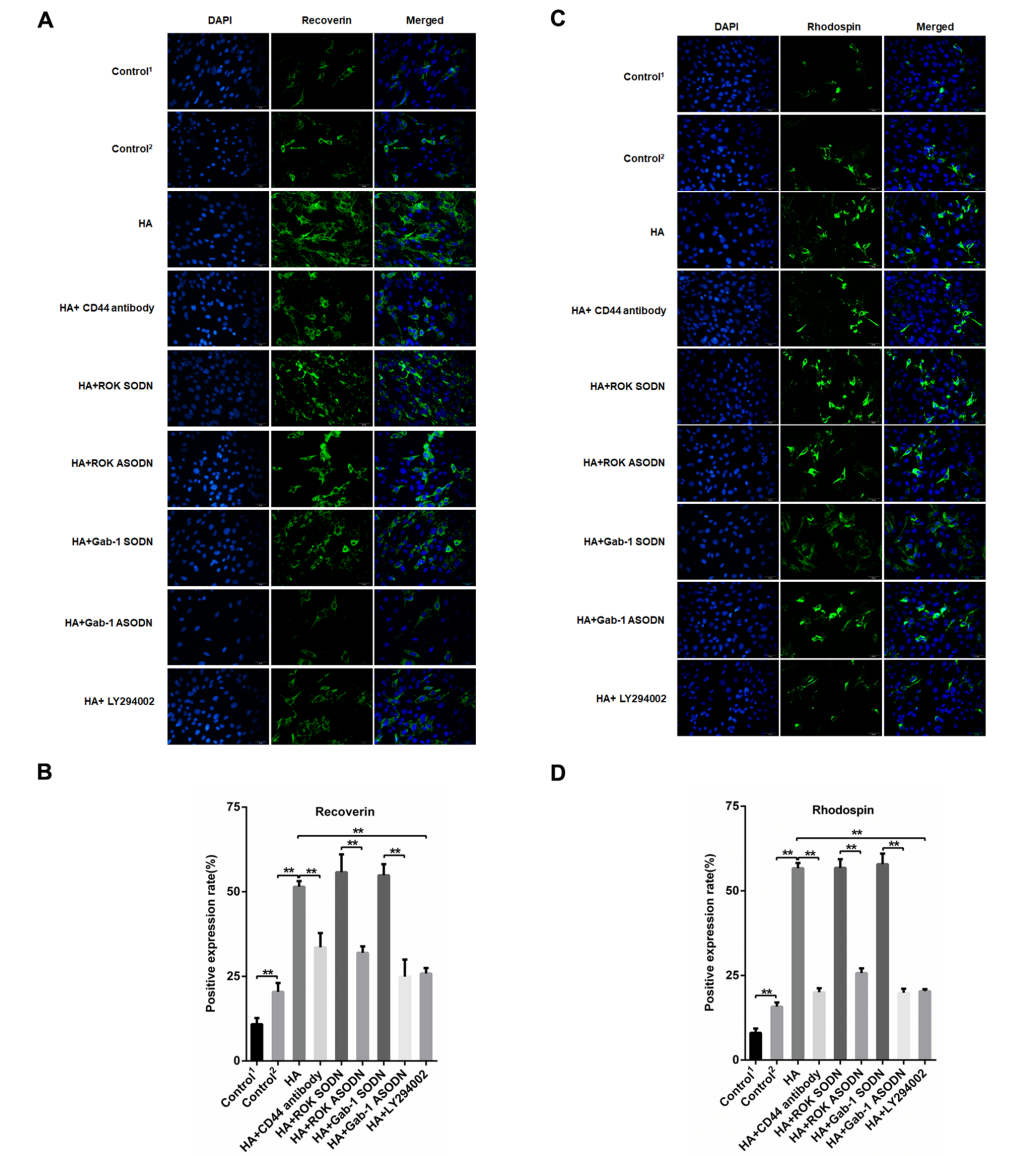
HA-CD44 interaction, ROK/Gab-1 and PI3K/AKT signalling enhance the differentiation of RPCs towards neuronal cells. **(A, B)** Immunocytochemistry analysis was used to detect recoverin-positive cells with different treatments (n = 3). **(C, D)** Immunocytochemistry analysis was used to detect rhodopsin-positive cells with different treatments (n = 3). The data are shown as the mean ± SD, **p < 0.01; Scale bar = 20 mm; Control^1^, proliferation medium, Control^2^, differentiation medium

We investigated the effects of HA-CD44 interaction on RPCs differentiation. Immunofluorescence analysis showed that RPCs differentiation markers, such as β-III-tubulin (the pan-neuronal marker), cone-rod homeobox (Crx, the cone-rod photoreceptor precursor and photoreceptor marker), recoverin (the cone and rod photoreceptor marker) and rhodopsin (the rod photoreceptor marker), were significantly increased in differentiation medium compared with proliferation medium (Figs. 6 and 7). When the RPCs were treated with HA, the levels of retinal neuronal cell markers (β-III-tubulin, Crx, recoverin, rhodopsin) were significantly increased (Figs. 6 and 7). Further analyses indicated that RPCs treated with anti-CD44 antibody, ROK-specific ASODN, Gab-1-specific ASODN or PI3K inhibitor LY294002 had the opposite effect in the presence of HA (Figs. 6 and 7). These results indicate that the HA-CD44 interaction enhances the differentiation of RPCs towards neuronal cells via ROK/Gab-1 and PI3K/AKT signalling.

**Fig. 8:**
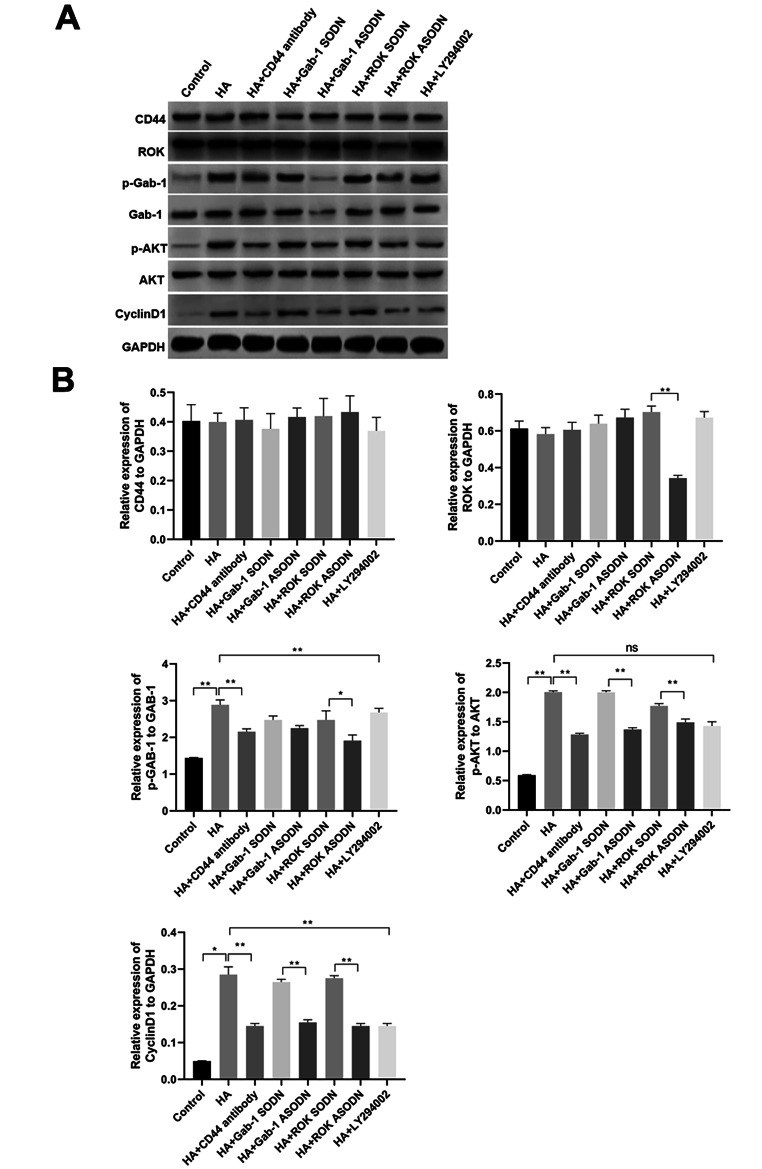
HA-CD44 interaction promotes RPCs proliferation via ROK/Gab-1 and PI3K/AKT signalling. **(A, B)** Expression levels of CD44, ROK, p- Gab-1, Gab-1, p-AKT, AKT and the proliferation marker cyclin D1 with different treatments were analysed by western blot (n = 3). The data are shown as the mean ± SD, *p < 0.05; ^**^p < 0.01

Based on the above results, we further explored whether an interaction occurs between ROK/Gab-1 and PI3K/AKT signalling in HA/CD44-mediated RPCs proliferation. Our results indicate that the protein expression levels of p-Gab-1, p-AKT and the proliferation marker cyclin D1 were increased significantly in RPCs treated with HA compared with the control or pretreated with anti-CD44 antibody followed by HA addition (Fig. 8A, B). Moreover, treatment of RPCs with ROK-specific ASODN resulted in downregulation of the protein expression of p-Gab-1, p-AKT and cyclin D1 in the presence of HA. Meanwhile, Gab-1-specific ASODN significantly reduced the protein expression of p-AKT and cyclin D1 in the presence of HA (Fig. 8A, B). Furthermore, the PI3K inhibitor LY294002 also greatly reduced the protein expression of p-AKT and cyclin D1 in the presence of HA but did not have a discernible effect on the expression of p-Gab-1 (Fig. 8A, B).

**Fig. 9:**
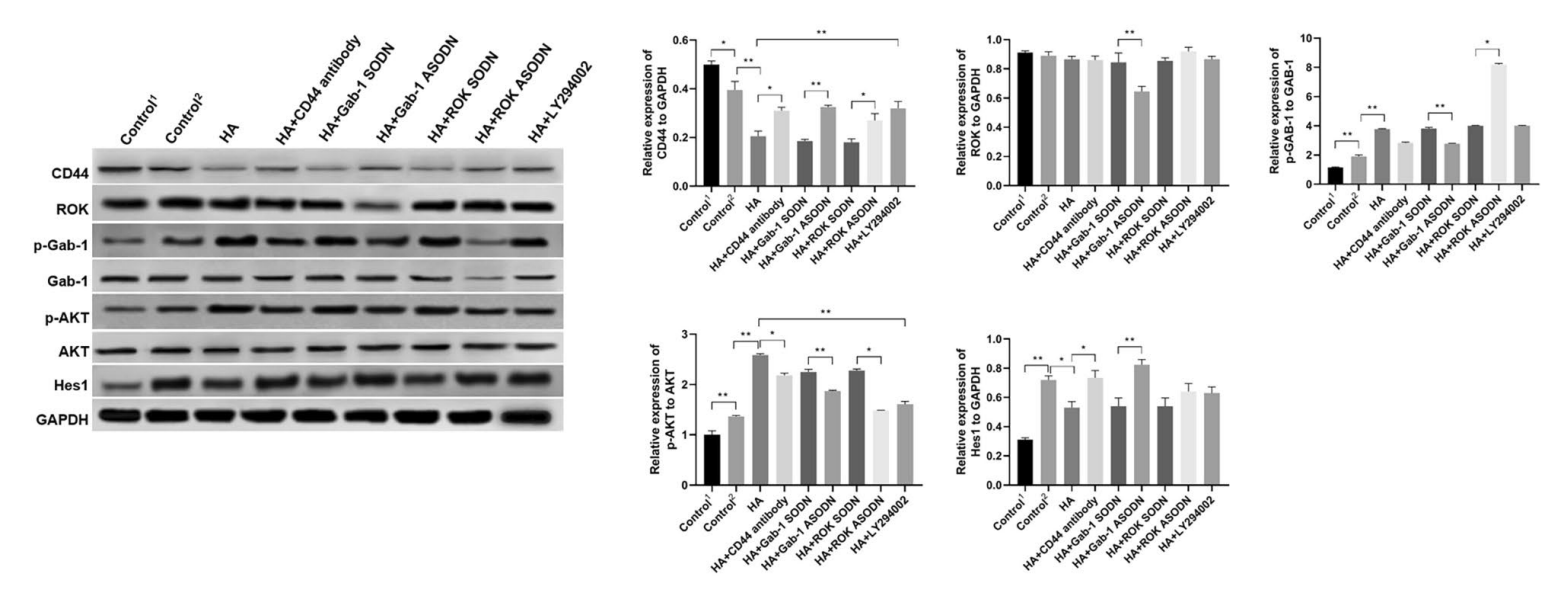
HA-CD44 interaction regulates the activation of the ROK/Gab-1/ PI3K/AKT axis. The expression levels of CD44, ROK, p- Gab-1, Gab-1, p-AKT, AKT and Hes1 with different treatments were analysed by western blot (n = 3). *p < 0.05; ^**^p < 0.01

We also investigated the potential interaction between ROK/Gab-1 and PI3K/AKT signalling in the HA/CD44-mediated RPCs neuronal differentiation process. Western blot analysis revealed that RPCs treated with differentiation medium demonstrated a significant increase in the expression of p-Gab-1, p-AKT and Hes1 compared to RPCs treated with proliferation medium (Fig. 9). However, the expression of the stem cell marker CD44 was significantly reduced (Fig. 9). Further analyses indicated that HA treatment significantly increased the expression of p-Gab-1 and p-AKT, and reduced the expression of CD44 and Hes1 (Fig. 9). Meanwhile, RPCs treated with anti-CD44 antibody had the opposite effect in the presence of HA (Fig. 9). Moreover, treatment of RPCs with ROK-specific ASODN significantly reduced the protein expression of p-Gab-1, and p-AKT and increased the expression of CD44 and Hes1 in the presence of HA (Fig. 9). Gab-1-specific ASODN significantly reduced the protein expression of p-AKT and increased the expression of CD44 and Hes1 in the presence of HA (Fig. 9). Furthermore, the PI3K inhibitor LY294002 also greatly reduced the protein expression of p-AKT and increased the expression of CD44 and Hes1 in the presence of HA, but had not discernible effect on the expression of p-Gab-1 (Fig. 9).

These observations indicate that ROK/Gab-1 signalling is an important upstream activator of PI3K/AKT signalling required for HA/CD44-mediated RPCs proliferation and neuronal differentiation.

## Discussion

Studies have now demonstrated that transplanted photoreceptor precursor cell suspensions in animal models of retinal degeneration can restore visual function, including using RPCs, ESCs or iPSC-derived cells [4]. However, the survival rate of trans-planted cells has been generally lower than 5% and reduces with time. The number of successfully ‘integrating’ cells can be increased by a variety of treatments to the host environment, modulation of glial cell hypertrophy/activation and matrix metalloprotease activity and using anti-apoptotic factors such as X-linked inhibitor of apoptosis protein (XIAP), chondrotinase ABC, immunosuppression or CRISPR-Cas9 gene editing technologies [4, 16, 23]. To achieve successful RPC-based transplantation therapy for RD, the high efficacy of cell proliferation, differentiation and migration is great of importance [8, 20]. It’s helpful to clarify the mechanisms of the cell differentiation and migration. HA not only serves as the primary ECM component but also acts as an active signalling molecule [13–15]. Studies have established that manipulating of HA concentrations or interactions can significantly alter the activities of signalling pathways that are involved in the regulation of cell behaviour and oncogenesis [12]. Previous work has shown the potential clinical utility of HA as a vitreous substitute [24]. Moreover, the combination of HA and gold nanoparticles could enhance the stability of the whole carrier and promote their distribution across ocular tissues to reach the retina, which is a promising delivery system for the treatment of intraocular neovascularization and other disorders [25]. CD44 is the main receptor for HA that influences cell proliferation, survival and motility, and is known to be relevant to neural stem cells (NSCs) expansion and differentiation [26]. However, research on the HA-CD44 interaction in RPCs is rather scarce. As the retina belongs to the central nervous system, RPCs are thought to possess multipotency similar to NSCs. Furthermore, CD44 was found to be highly expressed in RPCs in vitro in the present study. It is reasonable to hypothesize that the HA-CD44 interaction might have an effect on RPCs behaviour, such as cell migration, proliferation and differentiation. In the present study, we reported that the HA-CD44 interaction promotes the migration of RPCs, which might be due to the activation of PKC/Nanog/miR-21 signalling. Furthermore, our data suggested that the HA-CD44 interaction promoted RPCs proliferation and retinal neuronal differentiation by invoking the ROK/Gab-1 signalling pathway, and subsequently activating the PI3K/AKT signalling pathway.

The HA-CD44 interaction leads to numerous cellular responses, including those that involve tyrosine kinases, PKC, PI3K, and cytoskeletal components [15, 27]. The stem cell marker Nanog plays a key role in the self-renewal and maintenance of pluripotency in embryonic stem cells [28]. Disruption of the interaction between HA and CD44 in breast tumour cells has been shown to inhibit PKC-Nanog signalling mediated miR-21 production and suppress cell survival [12, 28]. Previous studies have also demonstrated that miR-21 is involved in the promotion of cell invasion, migration, and growth [28]. Consistent with these studies, our data demonstrated that PKC/Nanog/miR-21 signalling is involved in HA-CD44-mediated RPC migration. We found that HA could promote the migration of RPCs while perturbing the interaction between HA and CD44 or suppressing PKC/Nanog/miR-21 signalling activity had the opposite effect on RPC migration. Growing evidence supports that IAP proteins (e.g. survivin and XIAP), MDR1 and eIF4A positively modulate migration, invasion and metastasis [29]. In our study, we found that HA could enhance the expression of these proteins, but reduce the expression of PDCD4, which is closely linked to apoptosis and translation inhibition.

Our study also supports that the HA-CD44 interaction may promote RPCs proliferation and retinal neuronal differentiation. In our attempts to identify the potential cell signalling pathway, we noticed that ROK/Gab-1 and PI3K/AKT are the two main pathways for cell proliferation and differentiation, which have been reported for tumour cells and stem cells [30]. PI3K/AKT is known as an important proliferation-related signalling pathway as well as a differentiation-related signalling pathway in mesenchymal stem cells (MSCs) [31]. Moreover, a previous study demonstrated that ROK/Gab-1 signalling acts as the upstream effector of PI3K/AKT signalling during HA/CD44-mediated cell functions [30]. Most importantly, a recent study demonstrated that the Gel-HA hydrogel markedly enhanced RPCs proliferation, while RPCs cultured with the Gel-HA-PDA hydrogel might be programmed to differentiate into neurons by interaction with PI3K/AKT signalling [8]. Our results are consistent with this report showing that HA markedly upregulated the expression of the retinal progenitor-related marker nestin and the cell proliferation marker Ki-67 in RPCs in proliferation medium, and upregulated the expression of differentiation markers in differentiation medium. Furthermore, we found that ROK/Gab-1 signalling is an important upstream activator of PI3K/AKT signalling required for HA/CD44-mediated RPCs proliferation and neuronal differentiation. Tumorigenicity is also a concern when using RPCs; however, there was no evidence of uncontrolled cell growth, tumour formation or unexpected retinal structure alterations not only in vitro but also in vivo for murine or human RPCs [5, 11, 16, 32]. In summary, our data suggested that the HA-CD44 interaction allowed RPCs proliferation and retinal neuronal differentiation by invoking the ROK/Gab-1 signalling pathway, and subsequently activating the PI3K/AKT signalling pathway. In the meanwhile PKC/Nanog/miR-21 signalling is involved in HA-CD44-mediated RPCs migration.

## Conclusion

Our study provides novel insights into how the HA-CD44 interaction regulates RPCs migration, proliferation and differentiation in vitro. Further studies will focus on the underlying role of the HA-CD44 interaction in retinal development and its application in vivo for RD treatment.

### Electronic Supplementary Material

Below is the link to the electronic supplementary material.

Supplementary Material 1

## Data Availability

All the data used to support the findings of this study are included within the article and its supplementary material.
